# P-318. Clinical and genomic characterization of patients with methicillin-resistant *Staphylococcus aureus* horizontal transmission identified through prospective whole-genome sequencing: opportunities in the hospital and beyond

**DOI:** 10.1093/ofid/ofae631.521

**Published:** 2025-01-29

**Authors:** Rossana M Rosa, Gemma Rosello, Kelley Manzanillo, Renzo Cifuentes, Chris Ghaemmaghami, David Zambrana, Octavio Martinez, Lilian M Abbo

**Affiliations:** Jackson Health System , Fredericton, New Brunswick, Canada; Jackson Health System, Miami, Florida; Jackson Health System, Miami, Florida; University of Miami, Miami, Florida; Jackson Health System, Miami, Florida; Jackson Health System, Miami, Florida; Jackson Health System/University of Miami, Miami, FL; University of Miami Miller School of Medicine, Jackson Health System, Aventura, FL

## Abstract

**Background:**

We describe the clinical and epidemiological characteristics of patients involved in horizontal transmission detected through an Infection Prevention and Control (IPC) program integrating prospective whole-genome sequencing (WGS) of methicillin-resistant *Staphylococcus aureus* (MRSA) into routine activities.

**Figure:**
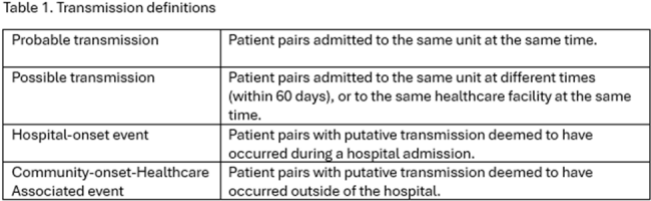

**Methods:**

Study conducted at a large county hospital in Miami, Florida between March 2023-January 2024. Clinical cultures of hospitalized patients underwent short-read sequencing. Pairs with < 25 SNPs were considered genomically related and investigated to determine the location and route of transmission. Cases were discussed by the IPC program with frontline healthcare workers and leaders to implement targeted measures where appropriate. Definitions are described in Table 1.

**Figure:**
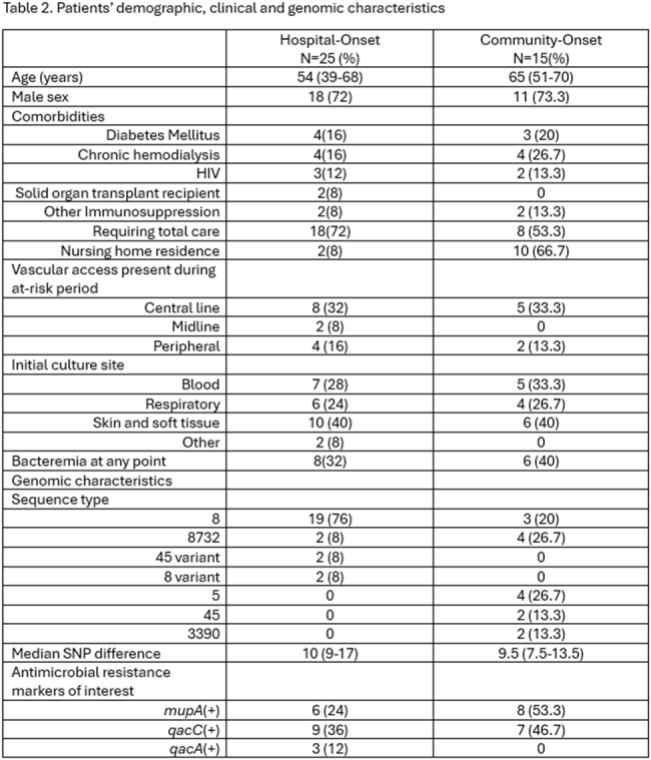

**Results:**

A total of 501 unique isolates were sequenced. We identified 21 putative transmission events, 9 probable and 12 possible, involving 40 patients. Transmission occurred in intensive care units (5), medical-surgical wards (3), hospital (without unit link, 5), nursing homes (4), jails (2) and in the community (2). Overall, 61.9% of events were hospital-onset (HO) and 8 (38.1%) were community-onset healthcare-associated (CO-HA).

Compared to HO cases, CO-HA cases were older, had higher proportions of diabetes, chronic hemodialysis, and were nursing home residents. Most HO cases belonged to sequence type (ST) 8 (76.0%), while CO-HA cases were more evenly divided among the STs identified (Table 2). Prevalence of carriage of *mupA* and *qacC* genes among CO-HA cases was higher than in HO cases at 53.3%/46.7% versus 24%/36% respectively.

There were 25 patients involved in HO transmission (12 index and 13 acquiring patients [1 patient linked to 2 others]). Six index cases were culture-positive on admission. Index cases who were culture negative on admission had a median number of days from admission to first positive culture of 28.4 (IQR 17.6-40.9). The median number of days overlapped between pairs was 6 (IQR 3-10) (Figure 1).

**Figure:**
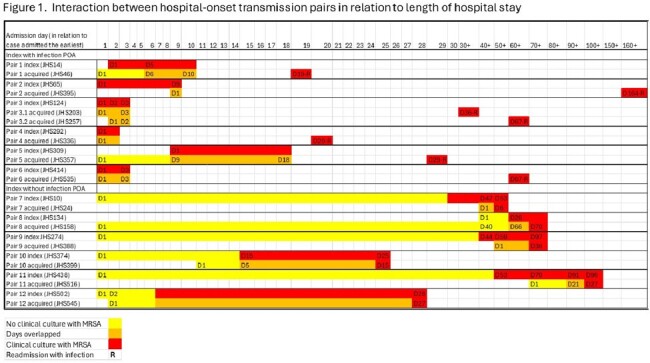

**Conclusion:**

Patients with CO-HA transmission of MRSA have a larger burden of chronic illness and antimicrobial resistance markers compared to HO cases. Mitigating the risk of MRSA infection requires efforts throughout the continuum of healthcare.

**Disclosures:**

**All Authors**: No reported disclosures

